# Critical Appraisal of Kinetic Calculation Methods Applied to Overlapping Multistep Reactions

**DOI:** 10.3390/molecules24122298

**Published:** 2019-06-21

**Authors:** Nikita V. Muravyev, Alla N. Pivkina, Nobuyoshi Koga

**Affiliations:** 1 Energetic Materials Laboratory, Semenov Institute of Chemical Physics, Russian Academy of Sciences, 119991 Moscow, Russia; alla_pivkina@mail.ru; 2 Chemistry Laboratory, Department of Science Education, Graduate School of Education, Hiroshima University, 1-1-1 Kagamiyama, Higashi-Hiroshima 739-8524, Japan; nkoga@hiroshima-u.ac.jp

**Keywords:** kinetic analysis, overlapping reactions, TG, DSC, kinetic deconvolution, isoconversional analysis, formal kinetic analysis

## Abstract

Thermal decomposition of solids often includes simultaneous occurrence of the overlapping processes with unequal contributions in a specific physical quantity variation during the overall reaction (e.g., the opposite effects of decomposition and evaporation on the caloric signal). Kinetic analysis for such reactions is not a straightforward, while the applicability of common kinetic calculation methods to the particular complex processes has to be justified. This study focused on the critical analysis of the available kinetic calculation methods applied to the mathematically simulated thermogravimetry (TG) and differential scanning calorimetry (DSC) data. Comparing the calculated kinetic parameters with true kinetic parameters (used to simulate the thermoanalytical curves), some caveats in the application of the Kissinger, isoconversional Friedman, Vyazovkin and Flynn–Wall–Ozawa methods, mathematical and kinetic deconvolution approaches and formal kinetic description were highlighted. The model-fitting approach using simultaneously TG and DSC data was found to be the most useful for the complex processes assumed in the study.

## 1. Introduction

Thermally-induced transformations in heterogeneous system usually do not obey the idealized single-step kinetic pattern but are comprised of consecutive or concurrent reaction steps. The reaction behavior is further complicated by the contribution of some physical phenomena (melting, diffusion, etc.) [1,2,3]. Thermal decomposition of solids represents a typical example of such complex process [4,5,6,7,8]. Determination of the kinetic parameters for the thermal decomposition is not a straightforward, but large experimental efforts should be paid for finding a possible way to attain the rigorous solution [9,10,11]. Alongside, examination of the applicability of different kinetic calculation methods through analyzing the mathematically simulated kinetic curves assuming the specific complex process is the possible method to obtain a guideline for the successful kinetic calculations [12,13,14]. For example, Svoboda et al. [15] analyzed the simulated process with independent reactions and revealed that the commonly used Kissinger method [16,17] provides the good estimate for the activation energy of the dominant reaction. Vyazovkin et al. applied isoconversional methods to the simulated parallel independent [18] and consecutive [19] reactions, and summarized the typical shapes of the activation energy dependency on the conversion degree [20]. Some examples of the complex reactions where the isoconversional hypothesis is not fulfilled were also reported [21,22,23]. Burnham [24] proposed the formal kinetic methods to describe the complex processes, but usually some preliminary insights are necessary to give the close initial guesses for many fitted parameters. The attempt to consider simultaneously various kinetic calculation methods to underline their advantages and shortcomings was performed by the Kinetic committee of ICTAC (International Confederation for Thermal Analysis and Calorimetry) [25]. Since then several thermokinetic techniques have been proposed [26,27], however, the critical assessment of the applicability of the modern thermokinetic methods for analyzing the complex processes is still missing.

If a partially overlapping multistep reaction is considered, the contributions of each component reaction step to the overall process determined by the different thermoanalytical (TA) techniques are not equal in general, because of the different physical quantities subjected to individual TA measurements. In some cases, the respective reaction steps can bring the opposite contributions to the overall process as measured using a TA technique. Experimental studies concerning the overall reaction composed of more than one reaction step with oppositely signed TA signals have been reported, i.e., partially overlapping mass loss and mass gain [3,28,29], or exothermic and endothermic events [30,31,32]. In practice, such complex processes are observed during thermal decomposition of energetic materials [5] and high-temperature operations with ionic liquids [33]. This particular situation, e.g., two overlapping mass-loss processes corresponding to endothermic and exothermic effects, respectively, educe that the apparent kinetic curves recorded using differential scanning calorimetry (DSC) can be different from that obtained using thermogravimetry (TG). The phenomenon is caused by different nature of these TA signals and was observed experimentally for isopropylammonium nitrate decomposition [30] and kerogen pyrolysis [34]. A sophisticated kinetic deconvolution technique have been applied recently to this type of solid-state reactions, where the kinetic parameters obtained for the constituted single processes have been linked with the physicochemical and physicogeometrical features of the transformations [31,32].

The aim of the present study is to evaluate the applicability of the available kinetic calculation methods to the simulated complex process characterized as the partially overlapping multistep reaction and to provide a guideline of successful kinetic analysis to the processes of this type. The kinetic curves were simulated mathematically (thus, correct kinetic parameters are available for verification), and processed using different kinetic calculation methods including Kissinger method [16], isoconversional methods [12,35,36,37], mathematical [27] and kinetic [31,38] deconvolution methods, and formal kinetic analysis [39,40]. Through the kinetic calculation using different methods, the resulting apparent kinetic parameters determined for simulated TG and DSC curves are mutually compared for finding the possible way to obtain relevant kinetic information of the real-life complex reaction process, for which one has limited information a priori. 

## 2. Theoretical

### 2.1. Simulation of Successive Reactions

The thermoanalytical data were simulated for the following theoretical process comprising two successive reaction steps:

(1) $$A(s)\overset{k_{1}}{\underset{\;}{\to }}B(s)+C(g),\;B(s)\overset{k_{2}}{\underset{\;}{\to }}D(s)+E(g)$$

To model the existence ratio of solid components – A(s), B(s), and D(s) at a time, a set of differential kinetic equations has to be solved. When TA signals under linear nonisothermal conditions at a heating rate *β* are simulated, a set of kinetic equations is expressed using conversion degrees, *α*_1_ and *α*_2_, for the respective component reaction steps [41]:

(2) $$\frac{d\alpha_{1}}{dT}=\frac{A_{1}}{\beta }e^{-E_{a1}/(RT)}f_{1}(\alpha_{1}),\;\frac{d\alpha_{2}}{dT}=\frac{A_{2}}{\beta }e^{-E_{a2}/(RT)}f_{2}(\alpha_{1},\alpha_{2}),\;$$

where *A* and *E*_a_ are the apparent Arrhenius parameters and *f*(*α*) is the kinetic model function. The subscripts 1 or 2 indicate the first and second component reaction steps. Considering the derivation of the kinetic rate data from derivative TG (DTG), the normalized overall reaction rate, (d*α*/d*t*)_DTG_, is expressed:

(3) $${\left(\frac{d\alpha }{dt}\right)}_{\mathrm{DTG}}=\eta \frac{d\alpha_{1}}{dt}+(1-\eta )\frac{d\alpha_{2}}{dt},\;$$

where *α* is the conversion degree for the overall reaction and *η* is the ratio of the total mass-loss value during the first reaction process, Δ*m*_1_, to the total mass-loss for the overall reaction, Δ*m*_Σ_: *η* = Δ*m*_1_/Δ*m*_Σ_. On the other hand, the normalized overall reaction rate derived from DSC, (d*α*/d*t*)_DSC_, is represented as follows [19]:

(4) $${\left(\frac{d\alpha }{dt}\right)}_{\mathrm{DSC}}=\gamma \frac{d\alpha_{1}}{dt}+(1-\gamma )\frac{d\alpha_{2}}{dt},\;$$

where *γ* is the ratio of the total thermal effect for the first reaction, *Q*_1_, to the total thermal effect for the overall reaction, *Q*_Σ_: *γ* = *Q*_1_/*Q*_Σ_. It must be noted from Equations (3) and (4) that DTG and DSC curves for the overall process are generally different because of different definitions of *η* and *γ*.

For simplicity, a series of kinetic curves were simulated mathematically by assuming a successive two first-order reactions; therefore, *f*_1_(*α*_1_) = 1 − *α*_1_ and *f*_2_(*α*_1_, *α*_2_) = *α*_1_ − *α*_2_. In addition, equivalent total mass loss for the first and second reaction processes was assumed, thus *η* = 0.5. The thermal effects of the component reaction processes were assumed to have opposite signs, and the absolute value of the total thermal effect of the first reaction process (exothermic) was set to be twice as large as that for the second reaction process (endothermic), i.e., *γ* = 2. Based on the aforementioned assumptions, four different cases of the temperature dependences of the rate constants of each component reaction step were simulated:

- Case 1: the rate constant for the first reaction, *k*_1_, is larger than that of the second reaction, *k*_2_, and this difference decreases with increasing temperature, *T*;
- Case 2: the value of *k*_1_ is larger than *k*_2_, and its ratio, *k*_1_/*k*_2_, is constant independent of *T*;
- Case 3: the value of *k*_1_ is smaller than *k*_2_, and this difference increases with *T*;
- Case 4: the value of *k*_1_ is smaller than *k*_2_, and *k*_1_/*k*_2_ is constant independent of *T*.

Variation of *k*_1_/*k*_2_ with temperature is plotted for cases 1–4 in Figure S1, and the apparent Arrhenius parameters assumed for simulating the kinetic curves are listed in Table 1. The simulation of the kinetic curves at different *β* values were performed using Mathcad (ver. 15.0), in which the fourth-order Senum and Yang approximation [42] was employed for the approximation of exponential temperature integral.

### 2.2. Kinetic Calculation Methods

Kinetic calculations for the mathematically simulated kinetic curves were performed using self-developed thermokinetic code THINKS [43], which comprises various calculation methods including Kissinger (ASTM E698 [17]), Friedman [35], Flynn–Wall–Ozawa (FWO) [36], Starink [44], and Vyazovkin [45] methods, combined kinetic analysis (CKA) [26], and model-fitting approaches, kinetic deconvolution [31,38] and formal kinetic analysis [39,40]. For analyzing the partially overlapping thermal events, the statistical peak separation technique, i.e., mathematical deconvolution [27], was applied. The fundamentals of these kinetic calculation methods can be found in original papers, the basic ideas relevant to the present study are implemented in the text.

## 3. Results and Discussion

### 3.1. Apparent Features of the Simulated Kinetic Datas

**Figure 1:**
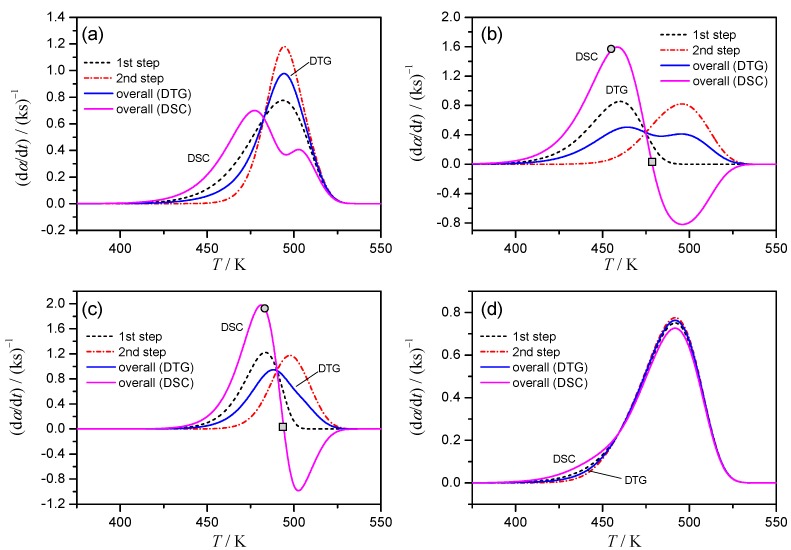
Comparison of the simulated kinetic curves at *β* = 2 K min^−1^ for each component reaction step calculated according to Equation (2), overall kinetic curve calculated according to Equation (3) (from DTG), and overall kinetic curve calculated according to Equation (4) (from DSC): (**a**) Case 1, (**b**) Case 2, (**c**) Case 3, and (**d**) Case 4.

The simulated kinetic curves at *β* = 2 K min^−1^ for the Cases 1–4 are shown in Figure 1. All the simulated kinetic curves at different *β* values for each case are presented in Figures S2–S5. Although various patterns of overlapping two mass-loss processes with exothermic and endothermic thermal effects are expected, the kinetic curves simulated by assuming four different cases in this study are believed to cover typical apparent features of the kinetic curves possibly obtained by TG and DSC measurements. 

In Case 1 (Figure 1a), the kinetic curve of DTG indicates a single peak, whereas overlapping two positive peaks with distinguishable peak tops are observed. At first glance, the process is misinterpreted as a single-step reaction from TG/DTG and as overlapping two exothermic reactions from DSC. However, the kinetic features assumed to simulate the kinetic rate can be deduced by comparing the kinetic curves derived from DTG and DSC: the peak top of DTG and the minimum between the two apparent positive peak tops in DSC appear at the same temperature. The assumed overlapping feature of the complex process is easily deduced in Case 2 (Figure 1b): DTG indicates overlapping of two peaks with well separated peak tops, while DSC indicates one positive and one negative peak. In Case 3 (Figure 1c), the smooth single peak is present on DTG curve. However, because of clearly separated positive and negative peaks on the kinetic curve of DSC, the assumed overlapping feature of the process is easily recognized. Case 4 is the most difficult one for deducing the complex features of the process from DTG and DSC data (Figure 1d). Because the kinetic curves derived from DTG and DSC are practically identical, it is the case usually interpreted from TG and DSC measurements as the single reaction process. In the present case, the situation appears as the result of the practically identical reaction temperature region and the same rate behavior assumed; however, similar situation is also expected when the contribution of a component reaction to the overall mass loss and thermal effect is very limited.

### 3.2. Kissinger Method 

For the kinetic analysis of the single-step reaction, the peak maximum of DTG and DSC curves, i.e., point where (d^2^*α*/d*t*^2^) = 0, contain important kinetic information [25] as formalized in the Kissinger method [16]: *E*_a_ value is evaluated from the shift of the peak temperature with varying *β* value. Because of its simplicity and robustness, the Kissinger method is one of the most widely used kinetic calculation methods for the determination of *E*_a_ value and also is implemented in ASTM E698-05 [17]. In general, application of the Kissinger method to the complex process as assumed in this study lacks theoretical validity. However, as demonstrated by Svoboda and Málek [15] for the kinetic analysis of the independent overlapping reactions, the results of Kissinger technique often reveal the relevant *E*_a_ value for the rate-limiting step.

Table 1 lists the apparent Arrhenius parameters for simulated kinetic data, calculated from the Kissinger plots and assuming the first-order reaction behavior. Irrespective of DTG and DSC data, superficial Arrhenius parameters were obtained for Case 1. Herein, the peak maximums cannot be correlated directly to the constituting single reactions as assumed by Kissinger method, but are determined by the superposition of two processes, which relative contributions are varied with *β* (Figure 1a, Figure S2). Table 1 also lists the variation range of the conversion degree at peak maximum, Δ*α*_p_, among the curves with different *β*. This variation should be negligible for rigorous application of Kissinger method [25], so its magnitude can serve as an indicator of the reaction complexity. In Case 2, both DTG and DSC data indicate well separated two peak maxima (Figure 1b) because of limited overlapping of two constituent reactions. The limited influence on each peak maximum from another stage results in nearly constant *α*_p_ with *β*, i.e., negligible Δ*α*_p_ (Table 1). Therefore, the Arrhenius parameters determined by Kissinger method for the first and second peaks of DTG and DSC data fully agree with the correct kinetic parameters. Application of Kissinger method in Case 3 brings the incorrect *A*, *E*_a_ values due to high overlapping of stages, that is recognized by a considerable Δ*α*_p_ values (Table 1). As was seen in Figure 1d, Case 4 is very specific with the same *E*_a_ values for stages, but lower rate constant *k*_1_ throughout the process. The Δ*α*_p_ values for DSC and DTG data are negligible supporting the value of Kissinger analysis. As a result, the *E*_a_ values obtained from DTG and DSC data closely correspond to that assumed to simulate the kinetic data. The preexponent value calculated from the overall DTG and DSC data correspond to the assumed *A* value for the first reaction step, because the first reaction step is the rate-limiting step in Case 4.

Summarizing the above findings, the Kissinger method becomes less applicable with increasing the interrelationship between the constituent processes. One should look on the magnitude of peak shift Δ*α*_p_ as a first indicator of the reaction complexity.

### 3.3. Isoconversional Methods

The kinetic analysis of the TA data using the isoconversional methods is recommended by the kinetic committee of ICTAC [25]. For the ideal single-step reaction recorded under ideal measurement conditions, a constant *E*_a_ value should be obtained at different *α* during the course of reaction, irrespective of the kind of isoconversional methods, i.e., integral method of Flynn-Wall-Ozawa [36], differential method of Friedman [35], and advanced integral method of Vyazovkin [45]. However, in practice, the constant *E*_a_ is often not attained even for the relatively simple processes, due to the gradual changes in reaction conditions, reaction geometry, and rate-limiting step as the reaction advances. Therefore, along with the examples of successful applications to complex processes [37], some superficial *E*_a_ values and observations of the kinetic parameters variation in an exotic manner during reaction course have been reported [22]. Moreover, when the isoconversional methods are applied to the multistep processes, the physical meaning of conversion degree *α* should be carefully reconsidered: while, in the fundamental kinetic equation, *α* is defined as the fractional reaction for the single reaction step [25], for the multistep process the *α* value is determined experimentally as the fraction of the total changes in the physical quantities during the overall process. Keeping in mind these theoretical limitations, several isoconversional methods were applied to the simulated kinetic data of Cases 1–4.

Application of differential Friedman [35] and integral FWO [36] methods reveals large (up to 35%) discrepancies of calculated *E*_a_ values for the same input data (Figure S6). A possible reason of inaccuracy of the Doyle approximation of temperature integral used in FWO method [25] was disregarded after coincidence of the FWO result with the output of Starink isoconversional method [44] that uses more precise representation of the temperature integral (Figures S6 and S7). Thus, the difference is inherent for the integral isoconversional methods and caused by considerable dependency of *E*_a_ on *α*, while in calculation the apparent *E*_a_ value is smoothed over 0–*α* region resulting in systematic error [45]. To prove this, we used the advanced integral method developed by Vyazovkin [45] that is based on the optimization of incremental change in *E*_a_ by iterative calculation over small *α* − (*α* + Δ*α*) and therefore eliminates the “smoothing” effect. Indeed, it reveals the kinetic parameters equal to the output of differential Friedman method (Figure S6).

**Figure 2:**
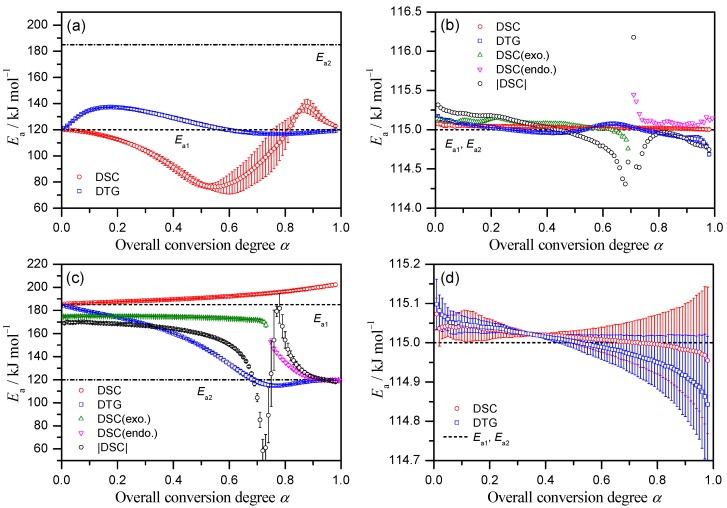
Isoconversional Friedman analysis of the simulated DTG and DSC data at *β* of 1–10 K min^−1^: (**a**) Case 1, (**b**) Case 2 (for clarity the uncertainties are not shown), (**c**) Case 3, and (**d**) Case 4.

The results of the isoconversional Friedman analysis applied to the simulated data of Cases 1–4 are shown in Figure 2 along with the assumed values for the constituting steps (dashed lines). For Case 1, the *E*_a_ value calculated from DTG indicates the correct value of *E*_a1_ at the beginning and increases toward *E*_a2_ in *α* range characterized by overlapping of two reaction processes but goes back to *E*_a1_ at the final stage (Figure 2a). As for isoconversional dependency built on DSC data, its variation is superficial with no meaning for the lowering of *E*_a_, although the values at the beginning and end of the reaction correspond to *E*_a1_ as in the DTG. 

For Case 2 that describe two partially overlapping processes with similar *E*_a_ values, the *E*_a_ values determined from DTG are close to the correct *E*_a_ and approximately constant during the course of reaction. Due to distinction between the exothermic and endothermic effects on DSC curve of Case 2, the conversion, derived normally as a partial area under curve, will not only increase but decrease as the reaction advances. This fact results in *α* value larger than unity or smaller than zero depending on the magnitude relation between the exothermic and endothermic effects (Points designated as circles in Figure 1b,c correspond to the moment when the total conversion, calculated from DSC, is equal to unity). Thus, “normal” isoconversional procedure will treat the truncated to 1 part of DSC data offering the partial and distorted kinetic information. One of possible empirical procedures to deal with the “α > 1” issue is the separate consideration of the exothermic and endothermic parts [46]. The breakpoint between two parts is where DSC signal changes its sign (denoted by square in Figure 1b). To put the isoconversional kinetic parameters on a single plot we adjust the first “exo“ part as 0..0.7 (= *Q*_exo_/(*Q*_exo_ + |*Q*_endo_|)) range of overall conversion, while the second “endo” part – to 0.7..1 region (Figure 2b). The results of the “separation” procedure correspond well to the isoconversional data calculated on DTG and the correct *E*_a_ for both stages. Another possible approach to the “α > 1” issue is representing of the overall *α* as the index of the reaction advancement calculated as the partial area under the absolute magnitude of DSC data. It should be noted that the procedure lacks theoretical validity, because the sum of the absolute values of exothermic and endothermic peak areas possibly changes accompanied by the changes of overlapping degree of the exothermic and endothermic processes with *β*. Even in such theoretically invalid situation, the apparent *E*_a_ values calculated from DSC of Case 2 are close to the correct *E*_a_ value (Figure 2b, |DSC| curve).

The results for DTG of Case 3 seem to be more relevant as the barriers for stages are different. Although DTG curve looks like a smooth single peak (Figure 1c), the isoconversional analysis catches the variation of *E*_a_ values during the process (Figure 2c). The calculated *E*_a_ values at the beginning and the end of the overall reaction indicate the correct *E*_a_ values for the first and second mass-loss processes, respectively. For DSC data the “α > 1” issue arises due to clear exothermic and endothermic parts of the heat flow. The separate isoconversional analysis of exo- and endothermic parts reveals the activation energy for the first stage about 175 kJ mol^−1^ (exact value is 185 kJ mol^−1^), for the second endothermic stage *E*_a_ value approaches to 120 kJ mol^−1^, which is the correct value. Treatment of the absolute magnitude of DSC data as in Case 2, seems doubtful due to significant change in the sum of absolute peak areas, i.e., at *β* = 10 K min^−1^ it is larger in 1.23 times than at *β* = 1 K min^−1^. However, the *E*_a_ values calculated from |DSC| data indicate the similar variation as the reaction advances with that from DTG (Figure 2c).

Case 4 represents two stages that are overlapping in a smooth single peak either in DSC or DTG (Figure 1d). Because the *E*_a_ values assumed for the first and second reaction steps are identical, the results of Friedman method represent the close correspondence to the correct *E*_a_ value during the course of reaction both for DTG and DSC data (Figure 2d). The multistep reaction behavior cannot be deduced from the TA curves and the results of isoconversional analysis in this instance.

These results show the strength of isoconversional technique in catching the change of *E*_a_ in course of reaction and its weakness in case of significant overlapping. To evaluate the isoconversional results further, we compare the prediction of thermal behavior based on isoconversional kinetic parameters with the output of the exact model. Indeed, the results of the isoconversional analysis appear as the mathematical representation of the process. They can be easily extended outside of the temperature region where the kinetic parameters have been determined, i.e., for prediction purposes (e.g., [47]). What are the limits of this procedure, and does the isoconversional parameters derived for considered complex process allow successful prediction? Answering these questions, we compare the model output (exact solution) for heating rates from 10^−9^ to 10^9^ K min^−1^ with the prediction based on isoconversional results (obtained with data at 1–10 K min^−1^ rates). In Table S1 the results of the isoconversional-based simulation and exact solution are compared in terms of the peak temperatures of the conversion rate, Figure S8 depicts the reaction profiles. For Cases 2 and 4, i.e., where the *E*_a_ values for both stages are the same, the isoconversional prediction based on TG data is fully consistent with the true outputs from the assumed model. Isoconversional results for Case 2, where the overall *α* calculated from DSC goes above unity, reveal that for 0–1 region the prediction is correct (e.g., Figure S8c). To account the rest of the process, we apply two modified approaches, i.e., analysis of separate exo- and endothermic parts, and absolute magnitude of DSC, both results in inaccurate prediction. For Cases 1 and 3 with more complex kinetic pattern the isoconversional technique gives the results differing from the exact output of the assumed model in terms of the peak temperatures up to 260 K using DSC data and 75 K using TG data. Therefore, in case of high reaction complexity, the predictions based on the isoconversional kinetic parameters have to be performed with great caution.

### 3.4. Mathematical Deconvolution and Subsequent Combined Kinetic Analysis

An alternative approach is to extract (deconvolute) the constituent peaks from the complex profile for their subsequent kinetic analysis. This approach is based on the mathematical deconvolution—the empirical peak separation by summing up mathematically fitted component peaks using a fitting function, *F*(*t*) [48]:

(5) $$\frac{d\alpha }{dt}=\sum_{i=1}^{N}F_{i}\left(t\right),\;$$

where *N* is the total number of component reaction steps. The fitting function with the asymmetric peak shape is generally recommended, e.g., Fraser–Suzuki function [27]:

(6) $$F\left(t\right)=a_{0}\cdot \exp\left(-\ln2{\left[\frac{\ln\left(1+2a_{3}\frac{t-a_{1}}{a_{2}}\right)}{a_{3}}\right]}^{2}\right),\;$$

where *a*_0_, *a*_1_, *a*_2_, *a*_3_ are the amplitude, position, half-width and asymmetry of the curve, respectively. This approach has been successfully applied for real-life complex processes [49,50].

**Figure 3:**
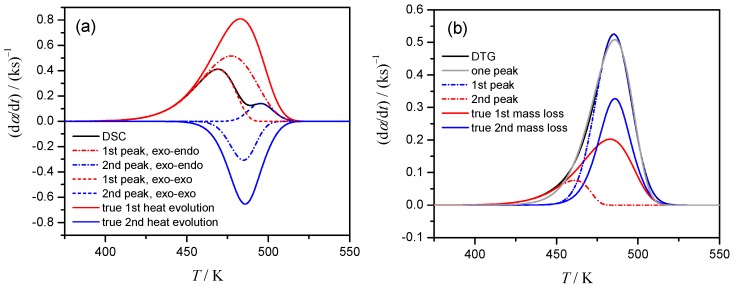
Selection of the peak combinations for Case 1, *β* = 1 K min^−1^: (**a**) superposition of two exothermic processes (dotted lines) or exothermic and endothermic ones (dashed lines) results in perfect correlation with DSC signal (black solid) but completely different from true heat evolution data, (**b**) DTG peaks are described well both by two peaks (dashed lines) and by single peak (dotted line), but both are different from true signals.

The deconvolution procedure for DSC data of Case 1, where the endothermic event is not apparent, yields the superficial results as shown in Figure 3a. The kinetic data derived from DSC (thick line) is equally well, with the correlation coefficient higher than 0.997, described by the superposition of two exothermic processes (dotted lines) or superposition of exothermic and endothermic events (dashed lines). Moreover, for the latter (correct choice) the deconvoluted exothermic and endothermic peaks are largely different from the assumed contribution of stages, i.e., *γ* (d*α*_1_*/*d*t*) and (1 − *γ*) (d*α*_2_*/*d*t*) (solid lines, Figure 3a). The problem of selecting the peak combination is easily eliminated for Cases 2 and 3, where the overall process clearly represents the combination of the exothermic and endothermic steps. Truly, in Case 2, where both stages have the same *E*_a_ value, but different *A* values, the deconvoluted peaks closely resemble the true reactions conversions (Figure S9a). However, in more realistic Case 3, when the *E*_a_ values for component reactions differ, the deconvoluted peaks although being exothermic and endothermic, not follow true reaction rates (Figure S9b). For DTG data, one is caught between deconvolution with two peaks (dashed lines, Figure 3b) or single peak (dotted line). However, the deconvoluted curves again are different from the true ones, i.e., *η*(d*α*_1_*/*d*t*) and (1 − *η*)(d*α*_2_*/*d*t*) (solid lines, Figure 3b). 

In the practical kinetic analysis, usually it is not possible to recognize the superficial mathematical deconvolution as was seen in Figure 3. For critical evaluation of the results we investigate how these superficial deconvoluted reactions for Cases 1–4 (Figure 3, Figure S9) behave being further subjected to the kinetic analysis. The kinetic analysis assumed a single-step reaction, so called combined kinetic analysis (CKA) [26], is based on the optimization of the linearized equation:

(7) $$\ln\frac{d\alpha }{dt}-\ln[\alpha^{m}{\left(1-\alpha \right)}^{n}]=\ln(cA)-\frac{E_{a}}{RT},\;$$

**Figure 4:**
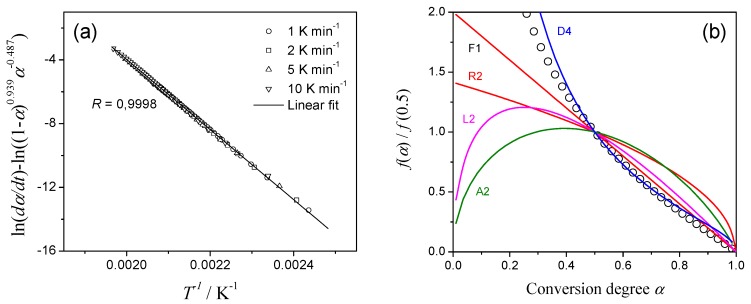
CKA analysis of the first reaction step of Case 1 deconvoluted from DSC curve by assuming two successive exothermic processes. (**a**) CKA plot, (**b**) Comparison of theoretical master plots of different kinetic models with calculated data (circles).

During the optimization we use for *m* and *n* the ranges of –1 ≤ *m* ≤ 2 and 0 ≤ *n* ≤ 2 as the reasonable limits [10]. The calculated linear dependency of Equation (7) is shown in Figure 4a for the first reaction in Case 1, deconvoluted as the combination of two exothermic processes on DSC (incorrect scheme!). Note, that the nice linearity not allows one to suspect the superficial nature of analyzed data. After the determination of the optimum *m* and *n* values, the reaction type have been discriminated by comparing the reduced *f*(*α*)/*f*(0.5) function with that for ideal reaction types. The analyzed reaction follows three-dimensional diffusion of Ginstling–Brounshtein model (designated as D4 in Figure 4b)!

Table S3 summarizes the apparent kinetic results obtained via the mathematical peak deconvolution and subsequent CKA treatment. Although the kinetic calculation using CKA method is superficially successful as was seen in Figure 4, the correct kinetic parameters were obtained only for Cases 2 and 4 with the same *E*_a_ for both component reactions. Friedman isoconversional analysis of the deconvoluted peaks (Figure S10) supports the conclusion: improper deconvolution disturbs the results, although some kinetic features of the initial kinetic model can still be noticed. Overall, the advantage of the mathematical deconvolution—high flexibility and excellent fit quality—appears as its drawback when the superficial peaks are reconstructed from the reaction profiles of the complex process.

### 3.5. Kinetic Deconvolution Analysis

Another deconvolution approach – kinetic deconvolution analysis (KDA) [10,48] –represents the input data as a superposition of the independent stages, *i*, with contributions *c_i_*:

(8) $$\frac{d\alpha }{dt}=\sum_{i}^{n}c_{i}A_{i}\exp(-\frac{E_{a,i}}{RT})f_{i}(\alpha_{i}).\;$$

An empirical reaction model *f*(*α*) is usually taken in a flexible Šesták–Berggren form [51]:

(9) $$f(\alpha )=\alpha^{m}\cdot {\left(1-\alpha \right)}^{n}\cdot {\left[-\ln(1-\alpha )\right]}^{p}.\;$$

Nonlinear regression is carried out for fitting the calculated data based on Equation (8) to the experimental data giving the optimized kinetic parameters *c_i_*, *E*_a_, *A*, *m*, *n*, *p* for each reaction step.

Whereas the mathematical deconvolution-based analysis and the KDA are based on the similar idea of superposition with several peaks, these two approaches are largely different in the light of methodological procedures. KDA accepts the “kinetic” shape of peaks according to Equations (8), (9), and this restriction results in more sound kinetic results (vide infra). However, the considerable number of optimized parameters imposes heavy demands on the reliability of the initial values for kinetic parameters. Therefore, the detailed kinetic information collected using a range of physicochemical and microscopic techniques is necessary to the successful KDA, in addition to preliminary kinetic calculations for kinetic curves recorded systematically [3,7,32]. 

To assess the value of the KDA for analyzed data and allow the nonlinear regression to converge, we take the Šesták-Berggren model in truncated form, i.e., with *p* = 0, and use the recently proposed approach for scheme of two partially overlapping reactions [31]. Therein, combining Equations (3) and (4), one obtains:

(10) $$P\left(T\right)=\frac{{\left(\frac{d\alpha }{dt}\right)}_{\mathrm{DSC}}}{{\left(\frac{d\alpha }{dt}\right)}_{\mathrm{DTG}}}=\frac{\gamma \frac{d\alpha_{1}}{dt}+\left(1-\gamma \right)\frac{d\alpha_{2}}{dt}}{\eta \frac{d\alpha_{1}}{dt}+\left(1-\eta \right)\frac{d\alpha_{2}}{dt}}.\;$$

**Figure 5:**
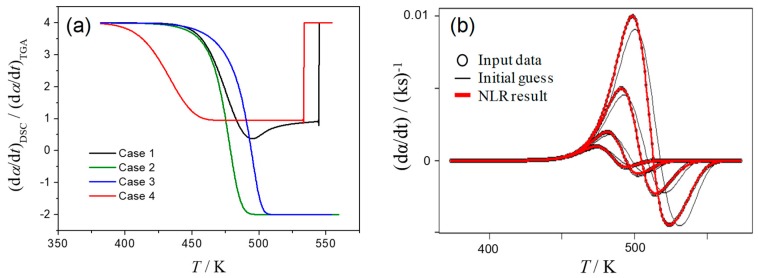
Kinetic deconvolution analysis: (**a**) Ratio of the conversion rates from DSC to DTG dependences on temperature for cases 1–4 at *β* = 2 K min^−1^, (**b**) Fit of DSC data for Case 3 under various heating rates (points) with Equation (8) with optimized parameters (thick red line).

Analyzing the *P*(*T*) dependency, we look at the beginning of the process, when d*α*_2_/d*t* ≈ 0 and *P* ≈ *γ*/*η*. In fact, in all Cases 1–4, the *P*(*T*) curve starts from value of 4 (Figure 5a). In Cases 2 and 3, where at the end of the process *k*_1_ exceeds *k*_2_, we obtain *P* ≈ (1 − *γ*)*/*(1 − *η*) = −2 since d*α*_1_*/*d*t* ≈ 0. Therefore, in specific instances when the contribution of either reaction step is negligible at the beginning and the end of the process, values of *γ* and *η* can be calculated from starting and final *P* values. These *γ* and *η* values were used as initial guesses in regression according to Equation (8).

Results of KDA show its advantage and robustness in revealing the correct reaction model, i.e., first-order reaction, using either DSC or DTG data (Table S3). As for kinetic pairs (*E*_a_, lg*A*) the correct results were obtained for simple Cases 2 and 4 where the ratio between the rate constants are independent on temperature. Considering the Cases 1 and 3, the statistical and visual (Figure 5b) quality of the fit is still high, but the *E*_a_ values differ from that in exact model. However, the *A* values compensate this difference in accordance with the kinetic compensation effect (KCE). It seems that this KCE is caused by the conceptual feature of the above deconvolution techniques: the conversion rate in form of Equation (8) in general is not equal to that parameter for the successive reactions due to unaccounted dependency of the component kinetic processes as considered in Equations (2) and (3) by the dependence of d*α*_2_/d*t* on *α*_1_ or in case of parallel processes because of the same interdependence of the conversion degrees for the components. 

### 3.6. Formal Kinetic Analysis 

The last considered method is formal kinetic analysis. It combines the strength of the model-fitting techniques with allowing for various connections between the process stages (e.g., successive, parallel and independent steps). This approach seems to be the most advantageous for the complex multistep process especially with exothermic and endothermic reactions in line with observations by Burnham [40]. The kinetic calculations have been performed with THINKS software by assuming single and various combinations of two-step reactions.

While performing the formal kinetic analysis, one faces with even higher (than for KDA) demands of the appropriate initial guesses and a need of the selection of the reaction scheme prior to calculation. Till now the selection of the reaction pattern depends on the experience of the researcher and the supplementary physicochemical insights to the process that are available. Recently, Muravyev and Pivkina propose to use the artificial neural networks to make a “recognition” of the kinetic scheme, however, the results only for the single-step reactions have been obtained [52]. Herein, we first tried the correct scheme with two subsequent first-order reactions to obtain the kinetic parameters and compare them with exact values. Overall, the kinetic parameters that were optimized throughout nonlinear regression based on DSC or DTG are close to that in assumed model (Table S4). But can we show that this kinetic scheme is indeed better than the above KDA model with two independent reactions, or model with the single-step reaction (e.g., for single DTG peak, Case 1)? From our calculations, all these schemes offer equally high correlation coefficients in certain cases and to select statistically best model we use the recently proposed advanced tool, the Bayes information criteria (BIC) [53,54]. Table 2 and Table S5 list the BIC values with the minimal one corresponding to the statistically best model. Evidently, true kinetic scheme can be identified by this kind of analysis. Also, we perform the calculations on joint DSC and DTG input data as was proposed recently by authors [31], i.e., the same reaction scheme but various contributions of stages for gravimetric and caloric data. Results show that using both datasets simultaneously increases the accuracy in kinetic parameters determination and allows easier distinguishing of the appropriate kinetic scheme (Figures S11 and S12).

High performance of the model-fitting analysis is evidenced by these results; however, the analyzed kinetic curves were drawn by assuming overlapping two-step reactions that are characterized by homogeneous-like first-order reactions with a simple interaction between the reaction steps as expressed by *f*_1_(*α*_1_) = 1 − *α*_1_ and *f*_2_(*α*_1_, *α*_2_) = *α*_1_ − *α*_2_. Further careful examinations with more complex input data are required to further assess the strength of the formal kinetic approach and its applicability to the real heterogeneous reactions. 

## 4. Summary and Outlook

**Figure 6:**
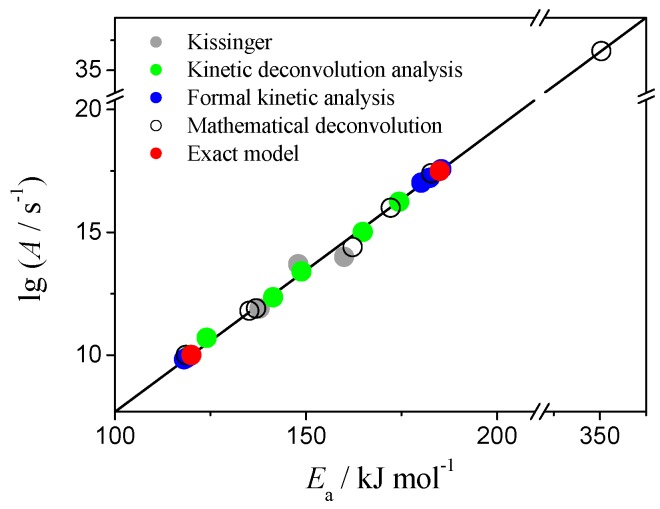
Kinetic compensation effect on the kinetic parameters calculated for Case 1 data.

The kinetic parameters calculated by above methods for both reaction steps of Case 1 are brought together in Figure 6 as lg*A* dependency on *E*_a_. The exact values (used to simulate the data) are marked as red circles on the plot. Results of Kissinger method, kinetic parameters for deconvoluted peaks and the output of the formal kinetic analysis fall onto the straight line drawn through the exact values of (lg*A*, *E*_a_). Same trend is observed for Case 3 (Figure S13). This phenomenon is known as kinetic compensation effect and it has been extensively discussed in literature [55,56,57]. Application of the incorrect reaction model *f*(*α*) or inappropriate computational technique to fit the data will manifest in results as KCE [57,58,59,60,61]. Apparently, our results shown in Figure 6 and Figure S13 reveal this, mathematical, type of KCE.

Summarizing the findings of present study, we can highlight the guidelines for designing the kinetic approach to the considered type of complex processes. First of all, it should be noted that the kinetic data resolved using TG and DSC are generally different for the multistep reactions and the observation of both signals is advantageous. Application of the isoconversional method can be useful for preliminary kinetic assessment as it often reflects some kinetic features of the process, e.g., the starting/final *E*_a_ values. However, the resulting changes of *E*_a_ during reaction course can easily become superficial when significant overlapping of stages takes place. The situation will propagate in isoconversional prediction; thus, it has to be performed with great precautions. The mathematical deconvolution technique shows high flexibility in catching of the reaction profile, while the flip side is extremely unstable kinetic results. Kinetic deconvolution analysis uses the same idea of superposition, but its “kinetic shape” of peaks increases robustness of the approach drastically. KDA method is recommended for the preliminary evaluation of the kinetic parameters, number of stages, and the reaction types. The formal kinetic analysis is shown to be the most useful in present evaluation since it provides the correct parameters for each reaction step in considered process. Its main problem is selection of an appropriate reaction scheme, must be solved in each particular case with the help of the preliminary kinetic analysis and additional physicochemical insights to the process. 

**Figure 7:**
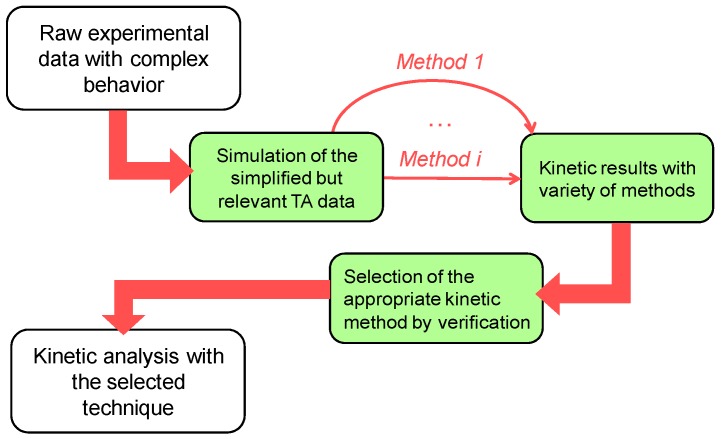
Proposed framework for the kinetic analysis of complex multistep processes.

There appears to be no single approach leading to the correct kinetic triplet in all situations but having the clear idea about the considered process and having analyzed the whole available experimental data (DSC and TG, structure changes, evolved gases etc.) will help one to select an appropriate way to perform the kinetic analysis and obtain correct results. As a general framework that eases the selection of particular kinetic calculation method, we use the following logical approach (Figure 7). The synthetic kinetic data that reveal the specific features of the real data was first generated. Then possible kinetic calculation methods are applied and justified by comparison of their output with exact kinetic parameters used in model. Last step is performing the kinetic analysis using selected kinetic calculation method over the real experimental data (i.e., with unknown answer). Present study and previous experimental work [31] taken altogether show the value of recommended approach.

## Figures and Tables

**Table 1:** Kinetic parameters calculated by the Kissinger plot for the simulated successive reactions of Cases 1–4.

Case	Step	Assumed Kinetic Parameters ^1^		*E*_a_/kJ mol^−1^^2^		lg(*A*/s^−1^) ^2^		Δ*α*_p_^3^	
		*E*_a_/kJ mol^−1^	lg(*A*/s^−1^)	DTG	DSC	DTG	DSC	DTG	DSC
1	1	120	10	138 ± 4	148 ± 1	12.9 ± 0.4	13.7 ± 0.1	0.06	0.19
	2	185	17.5	–	160 ± 10	–	14.0 ± 1.1	–	0.19
2	1	115	10.4	115 ± 1	115 ± 1	10.3 ± 0.1	10.4 ± 0.1	0.002	0.007
	2	115	9.4	115 ± 1	115 ± 1	9.4 ± 0.01	9.4 ± 0.1	0.002	0.003
3	1	185	17.5	203 ± 4	177 ± 2	19.3 ± 0.4	16.7 ± 0.3	0.11	0.11
	2	120	10	–	159 ± 3	–	14.0 ± 0.3	–	0.16
4	1	115	9.5	116 ± 3	116 ± 2	9.6 ± 0.3	9.6 ± 0.2	0.03	0.02
	2	115	11						

^1^ Stands for the kinetic parameters used in Equations (2)–(4) to calculate the input data. ^2^ Correlation coefficients of linear fit in Kissinger plot is higher than 0.999 for all cases. ^3^ Variation of *α*_p_ with *β* (1–10 K min^−1^).

**Table 2:** Bayes information criteria for the formal kinetic analysis of Case 1 data with several reaction schemes.

Kinetic Scheme	DSC Data	DTG Data	DSC+DTG Data
Single-step reaction	−4671	−6339	−23010
Two parallel reactions	−4888	−5355	−18186
Two consecutive reactions	−9873	−10733	−32343
Two independent reactions (KDA)	−6791	−7611	−24676

## Supplementary Materials

The following are available online, Figures S1–S13. Tables S1–S5.
