# Supplementary material for: Critical Appraisal of Kinetic Calculation Methods Applied to Overlapping Multistep Reactions
Source: Molecules. 2019 Jun 21;24(12):2298. doi: 10.3390/molecules24122298 (PMC6631284; doi:10.3390/molecules24122298)
Supplement: Supplementary file 1 [file molecules-24-02298-s001.pdf]

Critical appraisal of kinetic calculation methods applied to overlapping multistep reactions

Nikita V. Muravyev,[1,*] Alla N. Pivkina,[1] and Nobuyoshi Koga[2]

[1]*Semenov Institute of Chemical Physics, Russian Academy of Sciences, 4, Kosygin Str.. 119991 Moscow, Russia*

[2]*Chemistry Laboratory, Department of Science Education, Graduate School of Education, Hiroshima University, 1-1-1 Kagamiyama, Higashi-Hiroshima 739-8524, Japan*

Contents

Figure S1. Temperature dependence of the ratio of rate constants for the first and second reactions, k_1/k_2, assumed for simulating the kinetic rate data (cases 1–4). s2
Figure S2. Conversion rates for each reaction process calculated according to eq. (2), total conversion rate calculated according to eq. (3) (from DTG), and total conversion rate calculated according to eq. (4) (from DSC) for Case 1 at heating rates of 1 (a), 2 (b), 5 (c) and 10 K min^{-1}. s2
Figure S3. Conversion rates for each reaction process calculated according to eq. (2), total conversion rate calculated according to eq. (3) (from DTG), and total conversion rate calculated according to eq. (4) (from DSC) for Case 2 at heating rates of 1 (a), 2 (b), 5 (c) and 10 K min^{-1}. s3
Figure S4. Conversion rates for each reaction process calculated according to eq. (2), total conversion rate calculated according to eq. (3) (from DTG), and total conversion rate calculated according to eq. (4) (from DSC) for Case 3 at heating rates of 1 (a), 2 (b), 5 (c) and 10 K min^{-1}. s3
Figure S5. Conversion rates for each reaction process calculated according to eq. (2), total conversion rate calculated according to eq. (3) (from DTG), and total conversion rate calculated according to eq. (4) (from DSC) for Case 4 at heating rates of 1 (a), 2 (b), 5 (c) and 10 K min^{-1}. s4
Figure S6. Changes in E_a calculated from simulated TGA curves using different isoconversional methods of Friedman, Vyazovkin, Starink and Flynn-Wall-Ozawa: (a) Case 1, (b) Case 2, (c) Case 3, and (d) Case 4. .... s4
Figure S7. Errors in E_a calculated from simulated TGA and DSC curves using integral isoconversional methods: (a) Flynn-Wall-Ozawa- (FWO) method in comparison with differential Friedman method and (b) Comparison of FWO and Starink methods. s5
Figure S8. Comparison of the conversion-temperature curves calculated by isoconversional technique and exact outputs from assumed model: : (a) Case 1, heating rate of 10^{-9} K min^{-1}; (b) Case 1, 10^{9} K min^{-1}; (c) Case 2, heating rate of 10^{-9} K min^{-1}; (d) Case 2, 10^{6} K min^{-1}; (e) Case 3, heating rate of 10^{-9} K min^{-1}; (f) Case 3, 10^{9} K min^{-1}; (g) Case 4, heating rate of 10^{-9} K min^{-1}; (h) Case 4, 10^{9} K min^{-1} s6
Table S1. Comparison of the conversion rate peak temperatures for the assumed model and the isoconversional calculations s7
Figure S9. Deconvoluted using Fraser–Suzuki functions peaks along and the kinetic rate data from the exact model at β = 1 K min^{-1}: (a) DSC, Case 2, (b) DSC, Case 3, (c) DSC, Case 4 and (d) DTG, Case 4. .... s8
Figure S10. Isoconversional Friedman plots for the deconvoluted data: (a) DSC data, Case 1 (Figure 3a), (b) DTG data, Case 1 (Figure 3b), (c) DSC data, Case 2 (Figure S8a), (d) DSC data, Case 3 (Figure S8b). s8
Table S2. Results of the kinetic analysis on the deconvoluted peaks s9
Table S3. Summary of the results of the kinetic deconvolution analysis s10
Table S4. Summary of the results of the formal kinetic analysis with the kinetic scheme as two consecutive first-order reactions s11
Table S5. Bayes information criteria for the formal kinetic fit of case 3 data with several reaction schemes s11
Figure S11. Formal kinetic and KDA fit (red lines) of data for Case 1 (points). (a) fit of DSC data with two independent Bna reactions, (b) fit of DTG data with two independent Bna reactions, (c, d) fit of DSC and DTG data with two independent Bna reactions, (e, f) fit of DSC and DTG data with two consecutive Bna reactions. s12
Figure S12. Formal kinetic and KDA fit (red lines) of data for Case 3 (points). (a) fit of DSC data with two independent Bna reactions, (b) fit of DTG data with two independent Bna reactions, (c, d) fit of DSC and DTG data with two independent Bna reactions, (e, f) fit of DSC and DTG data with two consecutive Bna reactions. s13
Figure S13. Linear dependence between lg$A$ and E_a observed for the kinetic parameters calculated using various methods for the simulated kinetic rate data (Case 3). s13

[*] Correspondence: Tel\Fax: +74991378203. E-mail: n.v.muravyev@ya.ru

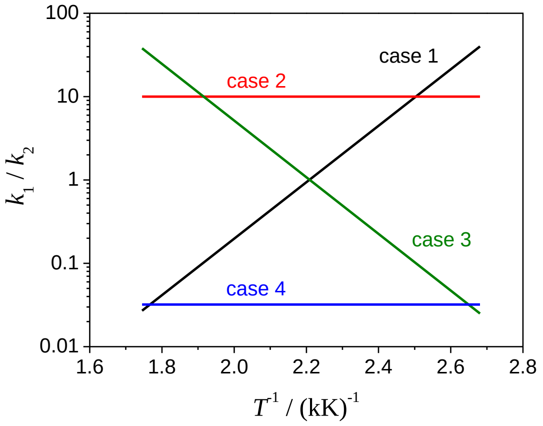

Figure S1. Temperature dependence of the ratio of rate constants for the first and second reactions, k_1/k_2, assumed for simulating the kinetic rate data (cases 1–4).

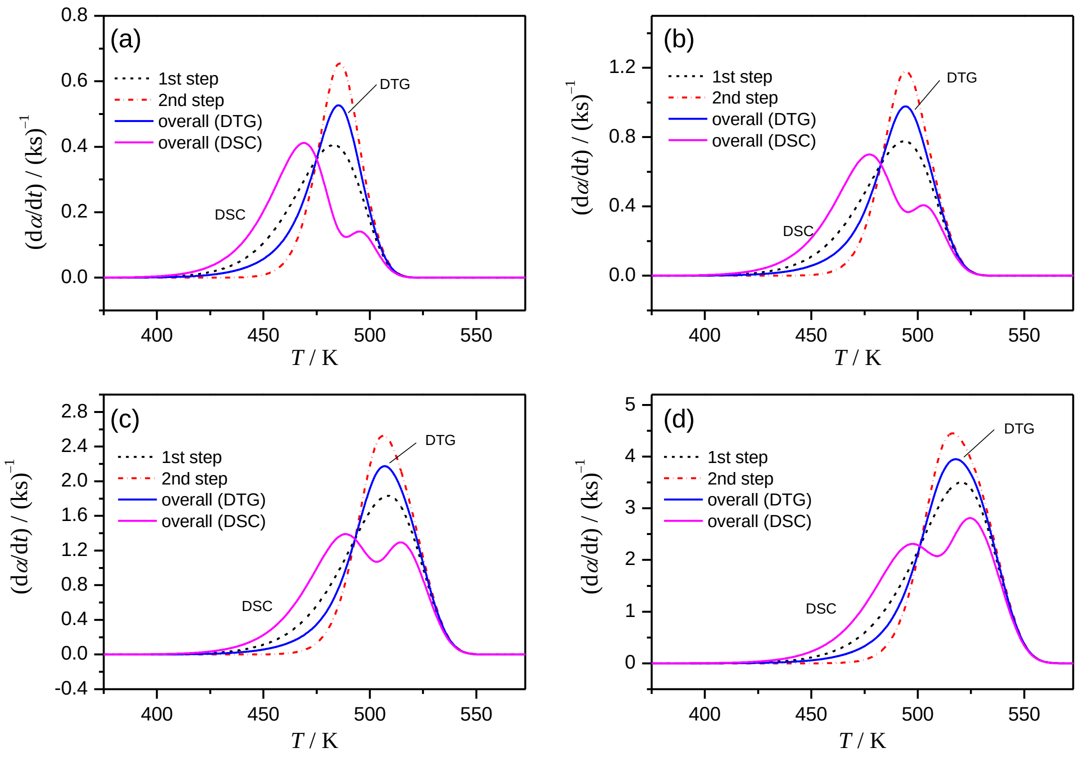

Figure S2. Conversion rates for each reaction process calculated according to eq. (2), total conversion rate calculated according to eq. (3) (from DTG), and total conversion rate calculated according to eq. (4) (from DSC) for Case 1 at heating rates of 1 (a), 2 (b), 5 (c) and 10 K min^{-1}.

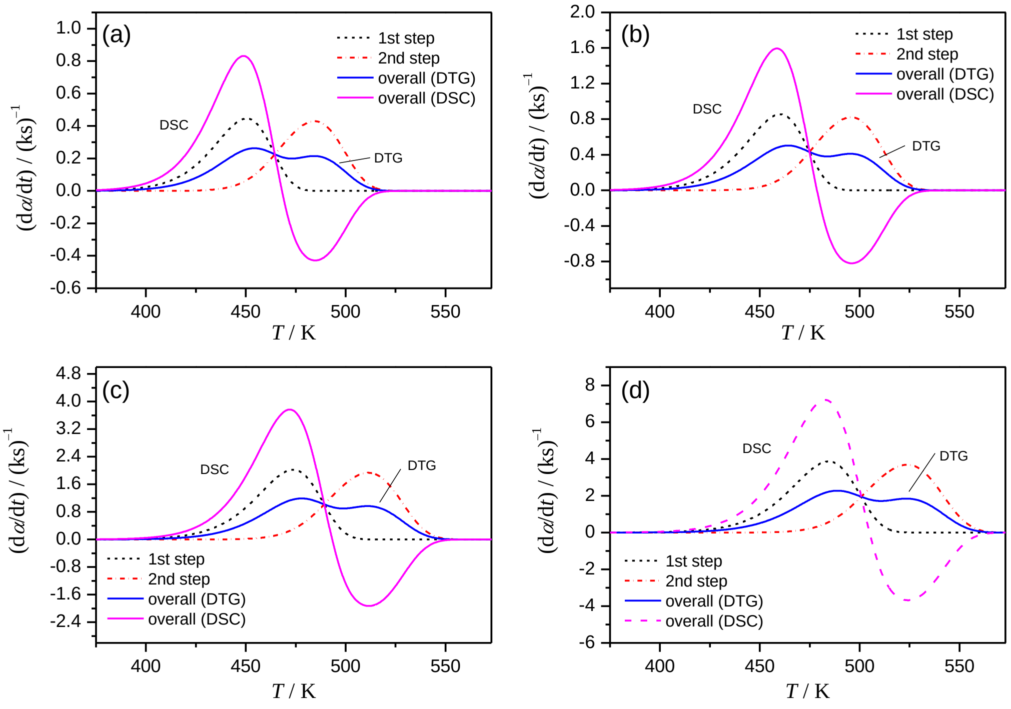

Figure S3. Conversion rates for each reaction process calculated according to eq. (2), total conversion rate calculated according to eq. (3) (from DTG), and total conversion rate calculated according to eq. (4) (from DSC) for Case 2 at heating rates of 1 (a), 2 (b), 5 (c) and 10 K min^{-1}.

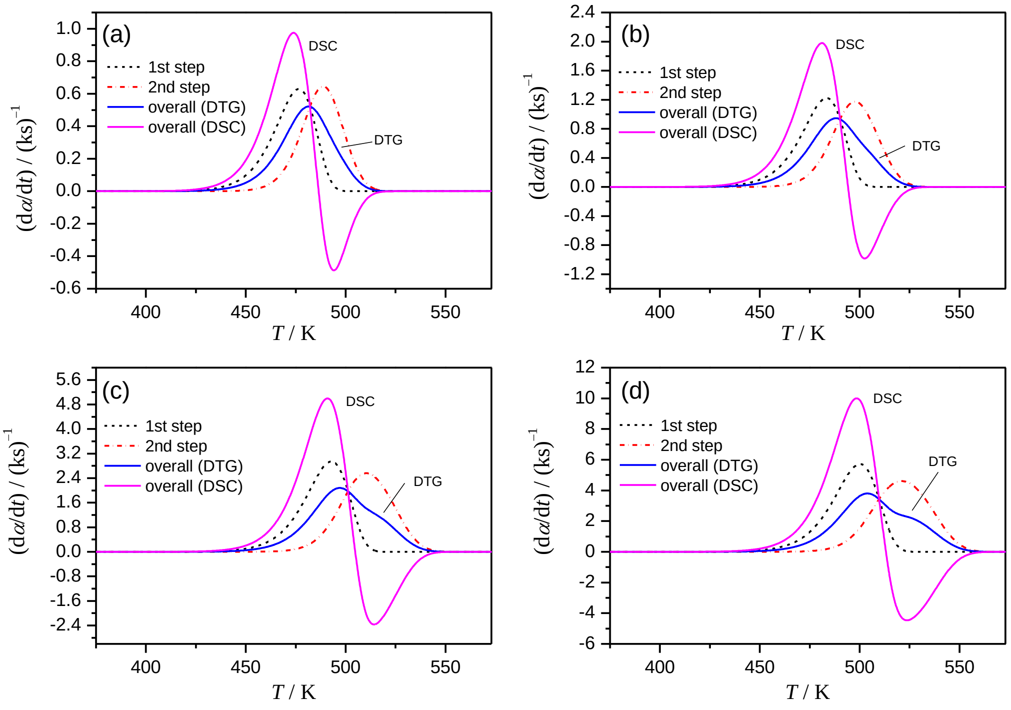

Figure S4. Conversion rates for each reaction process calculated according to eq. (2), total conversion rate calculated according to eq. (3) (from DTG), and total conversion rate calculated according to eq. (4) (from DSC) for Case 3 at heating rates of 1 (a), 2 (b), 5 (c) and 10 K min^{-1}.

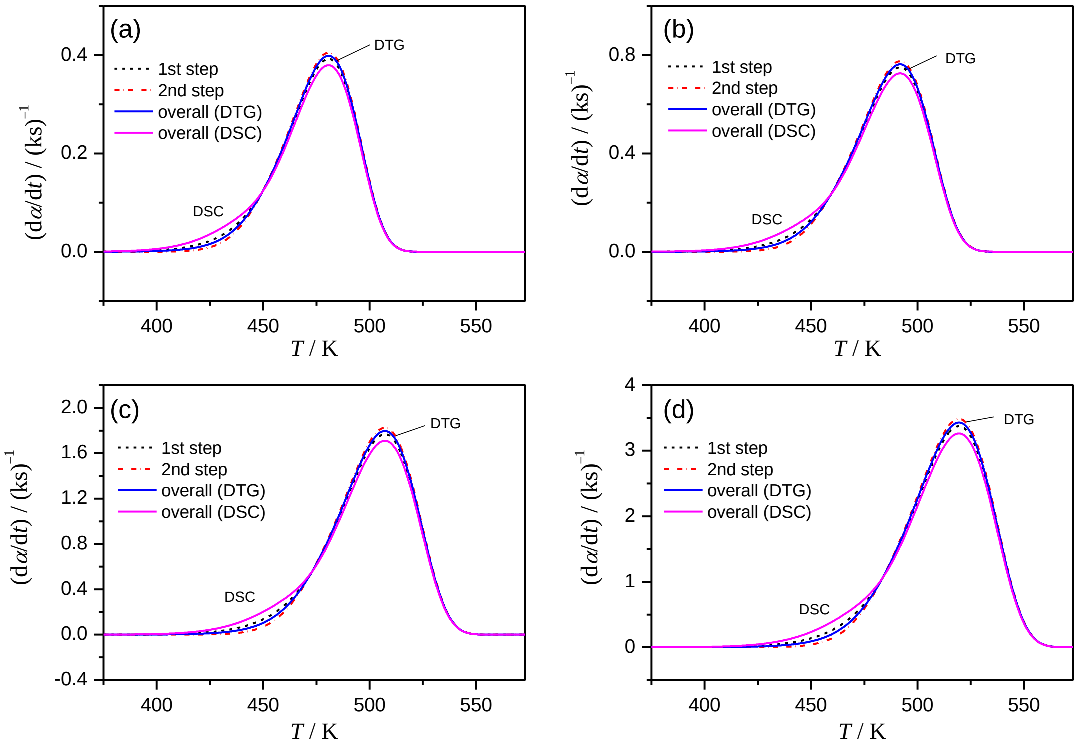

Figure S5. Conversion rates for each reaction process calculated according to eq. (2), total conversion rate calculated according to eq. (3) (from DTG), and total conversion rate calculated according to eq. (4) (from DSC) for Case 4 at heating rates of 1 (a), 2 (b), 5 (c) and 10 K min^{-1}.

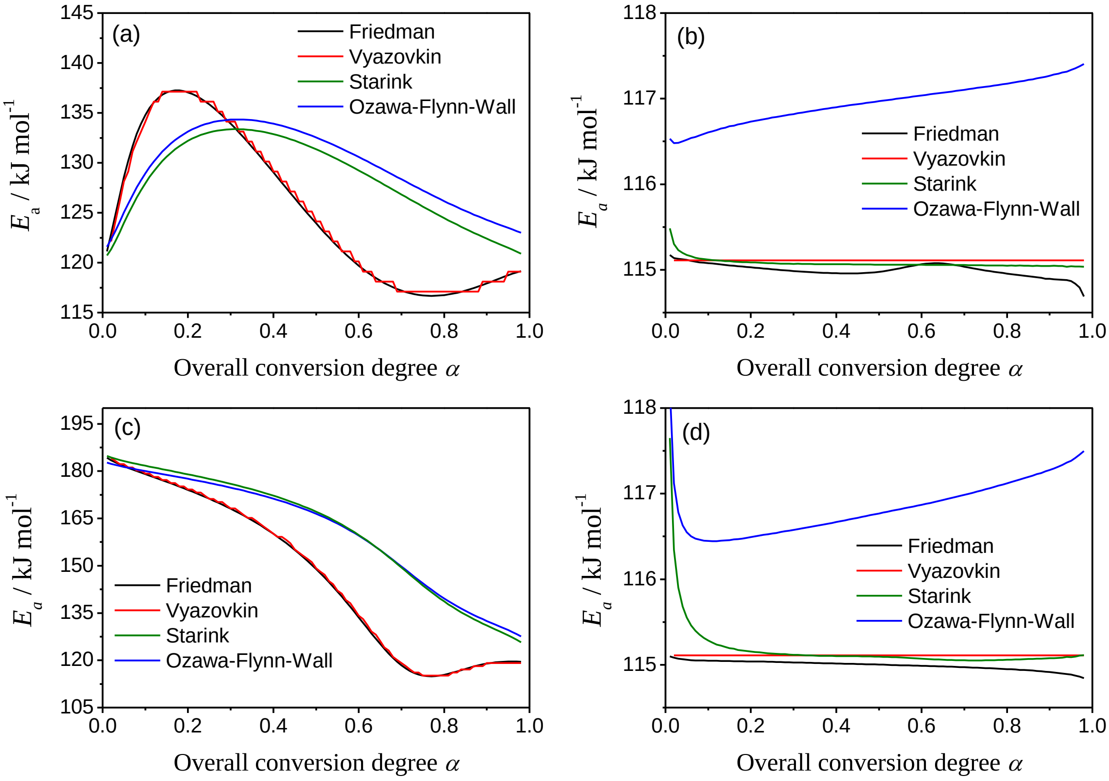

Figure S6. Changes in E_a calculated from simulated TGA curves using different isoconversional methods of Friedman, Vyazovkin, Starink and Flynn-Wall-Ozawa: (a) Case 1, (b) Case 2, (c) Case 3, and (d) Case 4.

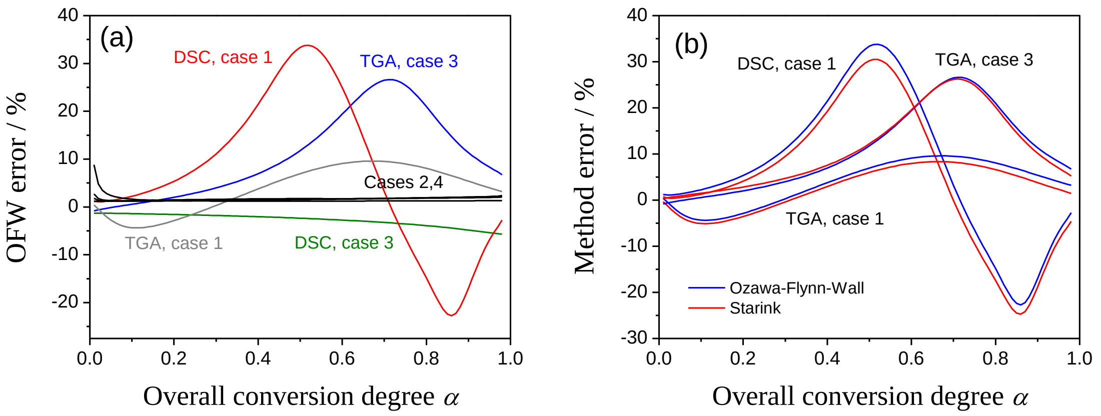

Figure S7. Errors in E_a calculated from simulated TGA and DSC curves using integral isoconversional methods: (a) Flynn-Wall-Ozawa- (FWO) method in comparison with differential Friedman method and (b) Comparison of FWO and Starink methods.

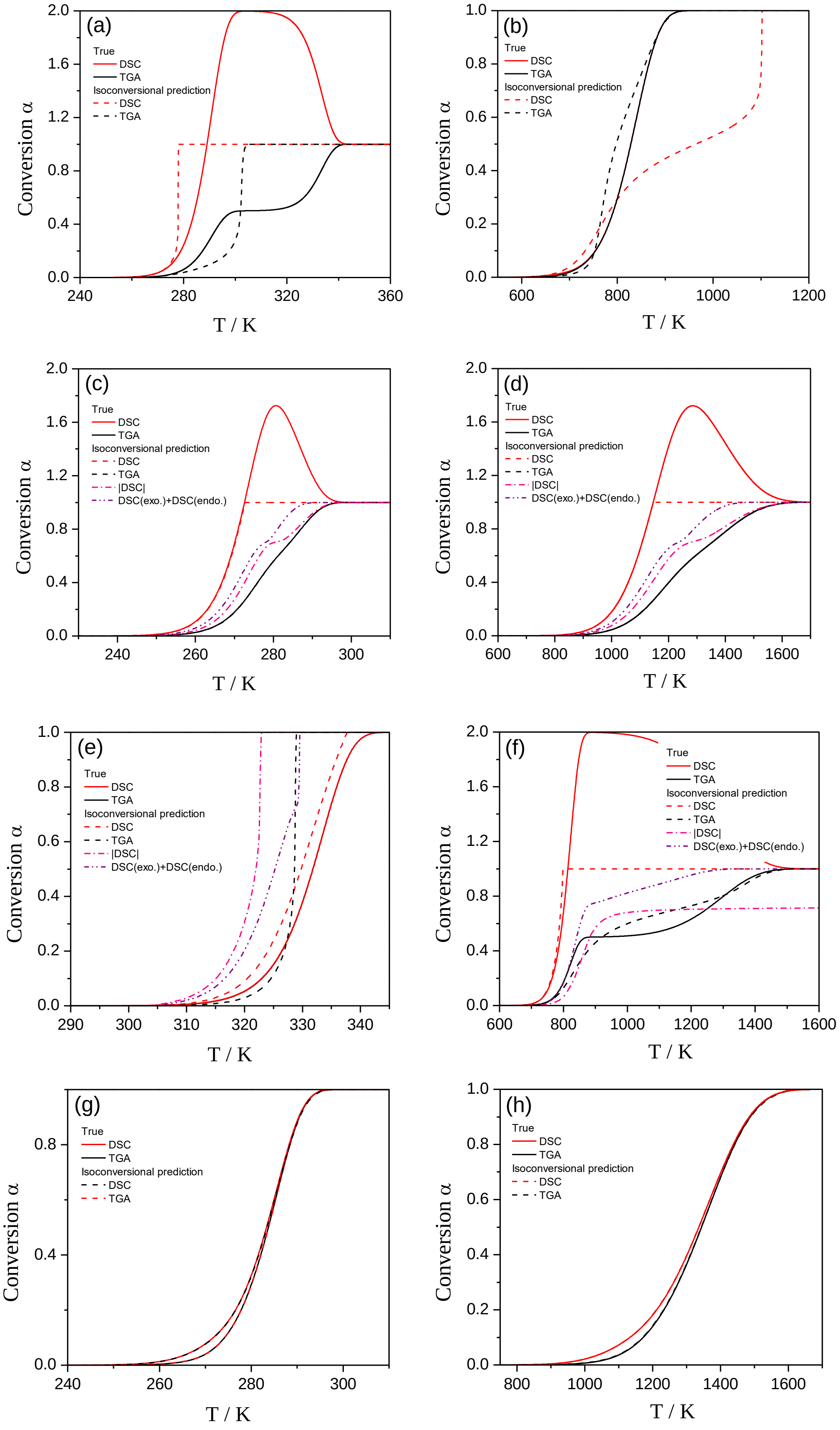

Figure S8. Comparison of the conversion-temperature curves calculated by isoconversional technique and exact outputs from assumed model: : (a) Case 1, heating rate of 10^{-9} K min^{-1}; (b) Case 1, 10^{9} K min^{-1}; (c) Case 2, heating rate of 10^{-9} K min^{-1}; (d) Case 2, 10^{6} K min^{-1}; (e) Case 3, heating rate of 10^{-9} K min^{-1}; (f) Case 3, 10^{9} K min^{-1}; (g) Case 4, heating rate of 10^{-9} K min^{-1}; (h) Case 4, 10^{9} K min^{-1}

Table S1. Comparison of the conversion rate peak temperatures for the assumed model and the isoconversional calculations

Case	signal	Peak Temperature [K] at rate [K min^{-1}]					
		1E-09	1E-06	0.001	1000	1E+06	1E+09
1	Exact model, DSC	291	336	395	615	839	*
	Isoconversional prediction, DSC	278	320	379	667	1102	2763
	Exact model, DTG	333	371	417	614	839	*
	Isoconversional prediction, DTG	303	346	405	591	766	1106
2	Exact model, DTG	276	318	374	576	781	1185
	Isoconversional prediction, DTG	276	318	374	576	781	1185
	Exact model, DSC	274	315	371	568	766	1154
	Isoconversional prediction, DSC	273	314	369	564	760	1144
	Isoconversional prediction, DSC-separate	272	313	367	560	752	1127
	Isoconversional prediction, DSC-absolute	274	315	371	568	765	1151
3	Exact model, DTG	334	371	418	556	663	822
	Isoconversional prediction, DTG	329	368	417	558	668	831
	Exact model, DSC	334	371	415	554	663	822
	Isoconversional prediction, DSC	332	368	415	549	651	797
	Isoconversional prediction, DSC-separate	329	369	419	552	666	835
	Isoconversional prediction, DSC-absolute	323	362	412	638	674	854
4	Exact model, DSC	286	331	392	618	859	1364
	Isoconversional prediction, DSC	286	331	393	618	857	1365
	Exact model, DTG	286	331	392	618	859	1364
	Isoconversional prediction, DTG	285	331	392	618	857	1362

*unstable solution

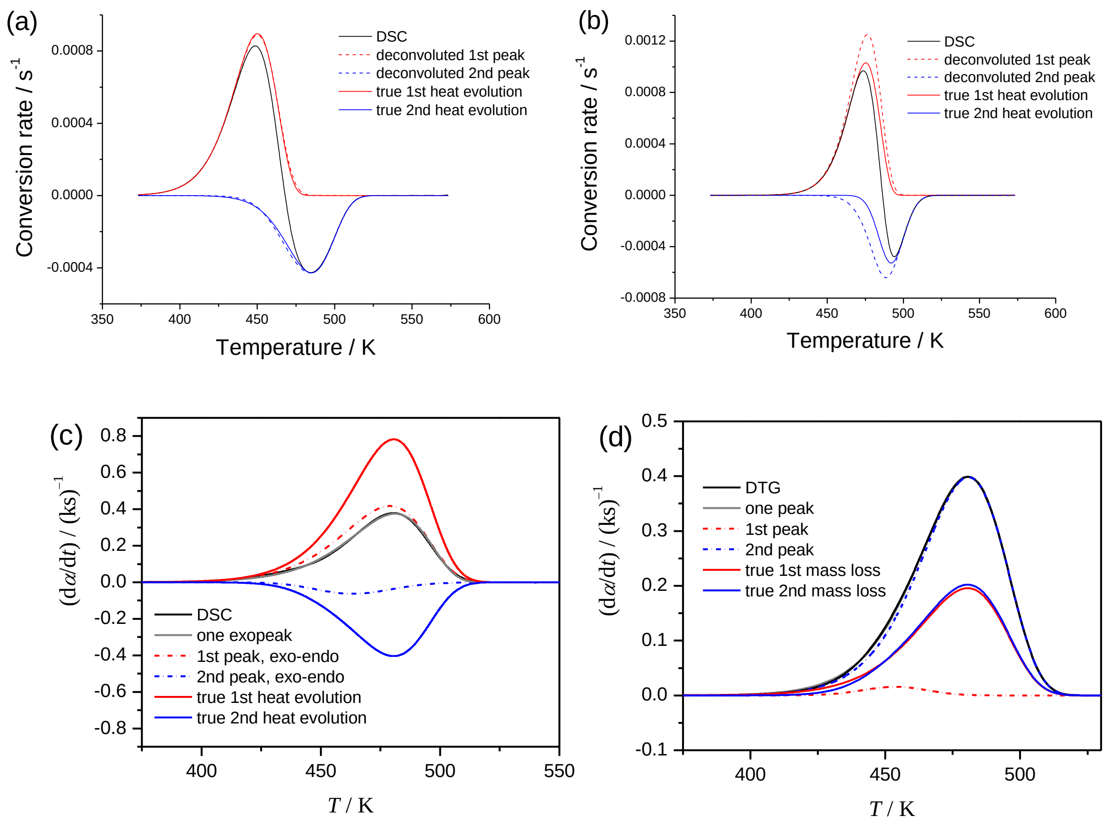

Figure S9. Deconvoluted using Fraser–Suzuki functions peaks along and the kinetic rate data from the exact model at $\beta = 1$ K min^{-1}: (a) DSC, Case 2, (b) DSC, Case 3, (c) DSC, Case 4 and (d) DTG, Case 4.

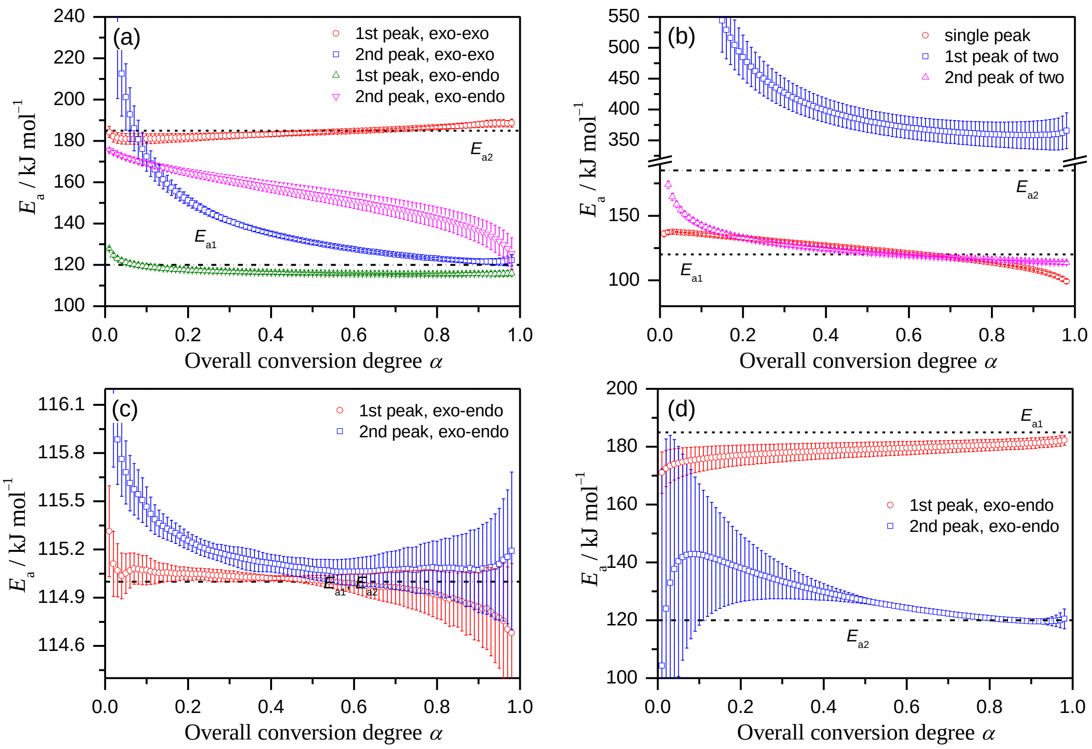

Figure S10. Isoconversional Friedman plots for the deconvoluted data: (a) DSC data, Case 1 (Figure 3a), (b) DTG data, Case 1 (Figure 3b), (c) DSC data, Case 2 (Figure S8a), (d) DSC data, Case 3 (Figure S8b).

Table S2. Results of the kinetic analysis on the deconvoluted peaks

Case	Signal	Peaks	First peak			Second peak			η	γ
			E_{a1} / kJ mol^{-1}	lg(A_1 /s^{-1})	*f*(α)	E_{a2} / kJ mol^{-1}	lg(A_2 /s^{-1})	*f*(α)		
1	DTG	2	350 ± 18	37.2 ± 2.0	*F3*	134 ± 4	11.5 ± 0.5	*F1*	0.07 ± 0.06	-
	DTG	1	135 ± 3	11.6 ± 0.4	*F1*	-		-	-	-
	DSC	2 (+, –) [a]	116 ± 2	9.7 ± 0.2	*F1*	169 ± 2	15.4 ± 0.2	*L2*	-	1.32 ± 0.03
	DSC	2 (+, +)	182 ± 4	17.5 ± 0.4	*D4*	162 ± 12	14 ± 1.3	*A3*	-	0.6 ± 0.2
2	DTG	2	115 ± 0.01	10.4 ± 0.001	*F1*	115 ± 0.03	9.4 ± 0.003	*F1*	0.45	-
	DSC	2 (+, –)	115 ± 0.07	10.4 ± 0.008	*F1*	115 ± 0.07	9.4 ± 0.008	*R3*	-	1.99
3	DTG	2	177 ± 6	16.6 ± 0.7	*F1*	148 ± 25	12.9 ± 2.7	*F1*	0.43 ± 0.08	-
	DTG	1	188 ± 2	17.5 ± 0.3	*F2*	-		-	-	-
	DSC	2 (+, –)	178 ± 6	16.7 ± 0.7	*F1*	147 ± 13	12.7 ± 1.4	*R3*	-	2.0 ± 0.2
4	DTG	2	115 ± 1.0	9.5 ± 0.1	*L2*	115 ± 0.1	10.3 ± 0.01	*F1*	0.03	-
	DTG	1	115 ± 0.03	9.5 ± 0.004	*F1*	-		-	-	-
	DSC	2 (+, –)	115 ± 0.1	9.5 ± 0.01	*F1*	115 ± 0.6	10 ± 0.07	*F1*	-	1.15
	DSC	1	115 ± 0.01	9.5 ± 0.001	*F1*	-		-	-	-

[a] Supposed exothermic peaks denoted as "+", endothermic as "–".

Table S3. Summary of the results of the kinetic deconvolution analysis

Input data: DSC								
	Case 1		Case 2		Case 3		Case 4	
Parameter	Value	± Error	Value	± Error	Value	± Error	Value	± Error
lg(A_1 /s^{-1})	10.7	0.2	10.4	0.0	16.8	0.1	9.4	0.1
E_{a1} / kJ mol^{-1}	124.0	1.5	115	0.0	177.9	0.4	115.0	0.7
n_1	1.21	0.01	1.03	0.00	1.00	0.00	0.96	0.01
m_1	0.06	0.01	0.00	0.00	0.01	0.00	-0.12	0.01
lg(A_2 /s^{-1})	13.4	0.2	9.4	0.0	11.3	0.1	10.0	0.7
E_{a2} / kJ mol^{-1}	148.8	2.0	115	0.1	132.6	0.5	115.8	6.4
n_2	1.22	0.02	1.02	0.00	1.10	0.01	2.00*	0.52
m_2	0.27	0.02	-0.01	0.00	0.14	0.02	0.86	0.07
γ	1.88	0.1	2.12	0.00	2.08	0.02	1.02*	0.01
Input data: DTG								
	Case 1		Case 2		Case 3		Case 4	
Parameter	Value	± Error	Value	± Error	Value	± Error	Value	± Error
lg(A_1 /s^{-1})	16.2	0.3	10.4	0.0	18.9	0.1	22.0	3.7
E_{a1} / kJ mol^{-1}	174.4	3.0	114.8	0.1	198.3	1.1	236	36
n_1	1.48	0.09	1.02	0.00	1.17	0.00	0.85	0.08
m_1	0.42	0.01	0.00	0.00	-0.03	0.00	-1.0	0.5
lg(A_2 /s^{-1})	10.0	0.1	9.4	0.0	11.0	0.0	9.5	0.2
E_{a2} / kJ mol^{-1}	119.6	0.8	114.7	0.1	129.7	0.4	114.5	1.5
n_2	1.03	0.01	1.02	0.00	1.09	0.00	1.03	0.02
m_2	0.15	0.01	-0.01	0.00	0.20	0.01	0.05	0.03
η	0.15	0.02	0.44	0.00	0.50	0.01	0.10*	0.02
Input data: DSC+DTG								
	Case 1		Case 2		Case 3		Case 4	
Parameter	Value	± Error	Value	± Error	Value	± Error	Value	± Error
lg(A_1 /s^{-1})	12.4	0.5	10.4	0.0	16.9	0.1	9.4	0.1
E_{a1} / kJ mol^{-1}	141.4	4.6	114.7	0.0	179.0	0.7	114.0	0.8
n_1	1.41	0.07	1.02	0.00	1.03	0.01	1.00	0.00
m_1	0.07	0.04	-0.00	0.00	0.02	0.00	0.02	0.01
lg(A_2 /s^{-1})	15.0	0.7	9.4	0.0	11.6	0.1	17.7	4.1
E_{a2} / kJ mol^{-1}	164.9	6.9	114.7	0.0	134.7	1.0	193.6	38.5
n_2	2*	0.14	1.02	0.00	1.22	0.02	2*	0.49
m_2	0.33	0.05	-0.00	0.00	0.22	0.02	-1*	0.34
γ	2.77	0.68	2.11	0.00	2.09	0.02	0.86	0.04
η	0.99*	0.13	0.44	0.00	0.45	0.01	0.99*	0.02

*limit during optimization

Table S4. Summary of the results of the formal kinetic analysis with the kinetic scheme as two consecutive first-order reactions

Input data: DSC								
Parameter	Case 1		Case 2		Case 3		Case 4	
	Value	± Error	Value	± Error	Value	± Error	Value	± Error
lg(A_1 /s^{-1})	9.9	0.0	10.3	0.0	17.2	0.0	9.4	0.0
E_{a1} / kJ mol^{-1}	118.7	0.3	113.7	0.1	182.0	0.1	114.0	0.2
lg(A_2 /s^{-1})	17.0	0.1	9.4	0.0	10.2	0.1	9.6	0.1
E_{a2} / kJ mol^{-1}	180.2	0.6	115.1	0.2	121.6	0.5	103.5	1.4
γ	1.99	0.02	1.98	0.00	1.96	0.00	1.73	0.03
Input data: DTG								
Parameter	Case 1		Case 2		Case 3		Case 4	
	Value	± Error	Value	± Error	Value	± Error	Value	± Error
lg(A_1 /s^{-1})	9.8	0.0	10.2	0.0	17.2	0.0	10.9	0.2
E_{a1} / kJ mol^{-1}	118.1	0.3	113.4	0.1	181.8	0.2	114.7	2.0
lg(A_2 /s^{-1})	17.6	0.2	9.2	0.0	9.8	0.0	9.3	0.0
E_{a2} / kJ mol^{-1}	185.4	1.5	112.5	0.1	118.0	0.3	113.2	0.1
η	0.51	0.01	0.50	0.00	0.50	0.00	0.02	0.00
Input data: DSC+DTG								
Parameter	Case 1		Case 2		Case 3		Case 4	
	Value	± Error	Value	± Error	Value	± Error	Value	± Error
lg(A_1 /s^{-1})	10.0	0.0	10.3	0.0	17.4	0.0	11.2	0.1
E_{a1} / kJ mol^{-1}	119.8	0.1	114.3	0.0	183.7	0.1	117.0	0.5
lg(A_2 /s^{-1})	17.2	0.0	9.3	0.0	9.8	0.0	9.4	0.0
E_{a2} / kJ mol^{-1}	182.4	0.3	114.5	0.1	118.4	0.2	114.3	0.0
γ	2.05	0.01	1.98	0.00	1.97	0.00	0.07	0.00
η	0.52	0.00	0.50	0.00	0.50	0.00	0.02	0.00

Table S5. Bayes information criteria for the formal kinetic fit of case 3 data with several reaction schemes

Kinetic scheme	DSC data	DTG data	DSC+DTG data
Single-step reaction	-8757	-12626	-18090
Two parallel reactions	-8689	-12606	-17920
Two consecutive reactions	-18399	-19326	-36714
Two independent reactions (KDA)	-15326	-16331	-28324

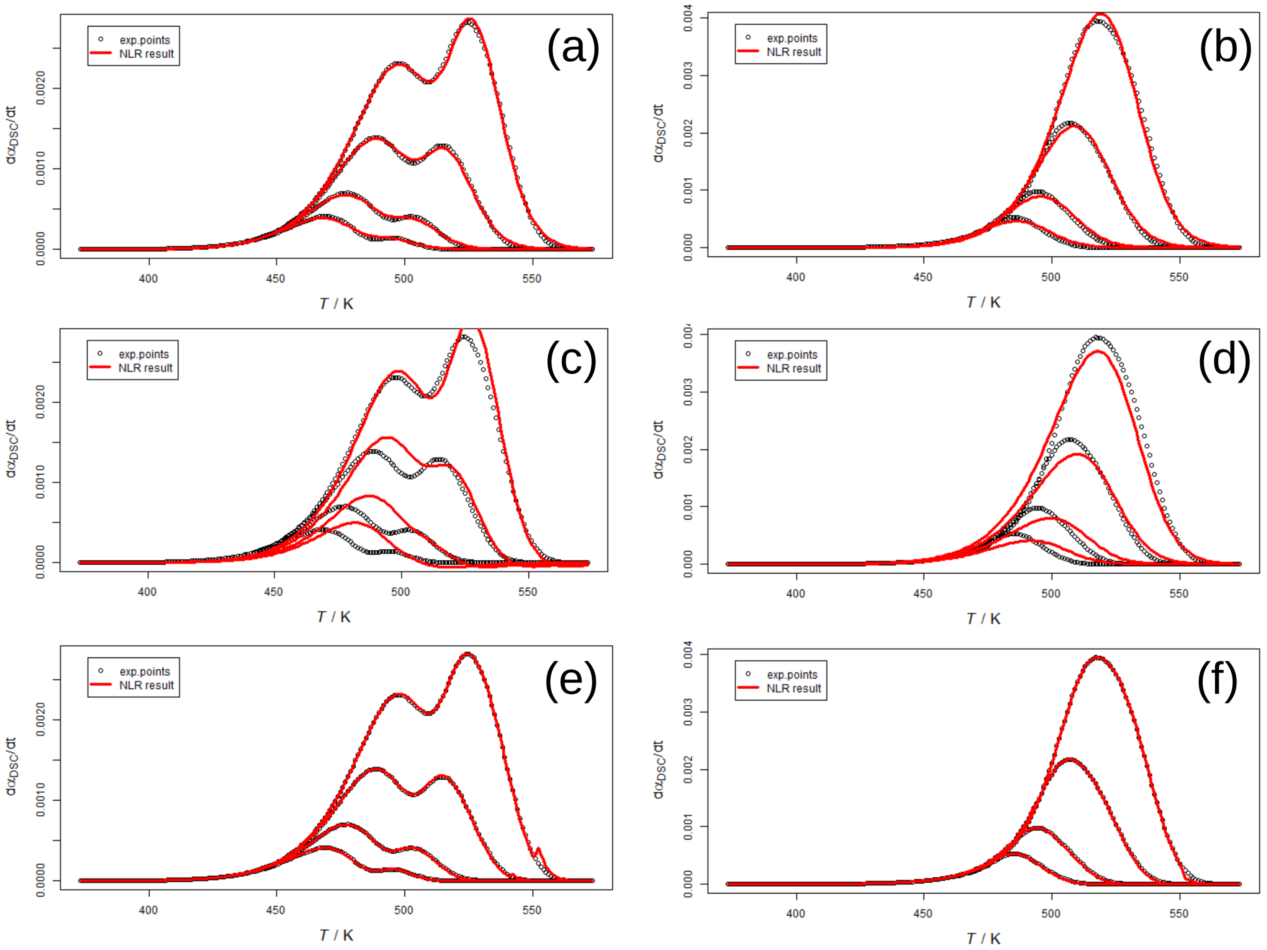

Figure S11. Formal kinetic and KDA fit (red lines) of data for Case 1 (points). (a) fit of DSC data with two independent Bna reactions, (b) fit of DTG data with two independent Bna reactions, (c, d) fit of DSC and DTG data with two independent Bna reactions, (e, f) fit of DSC and DTG data with two consecutive Bna reactions.

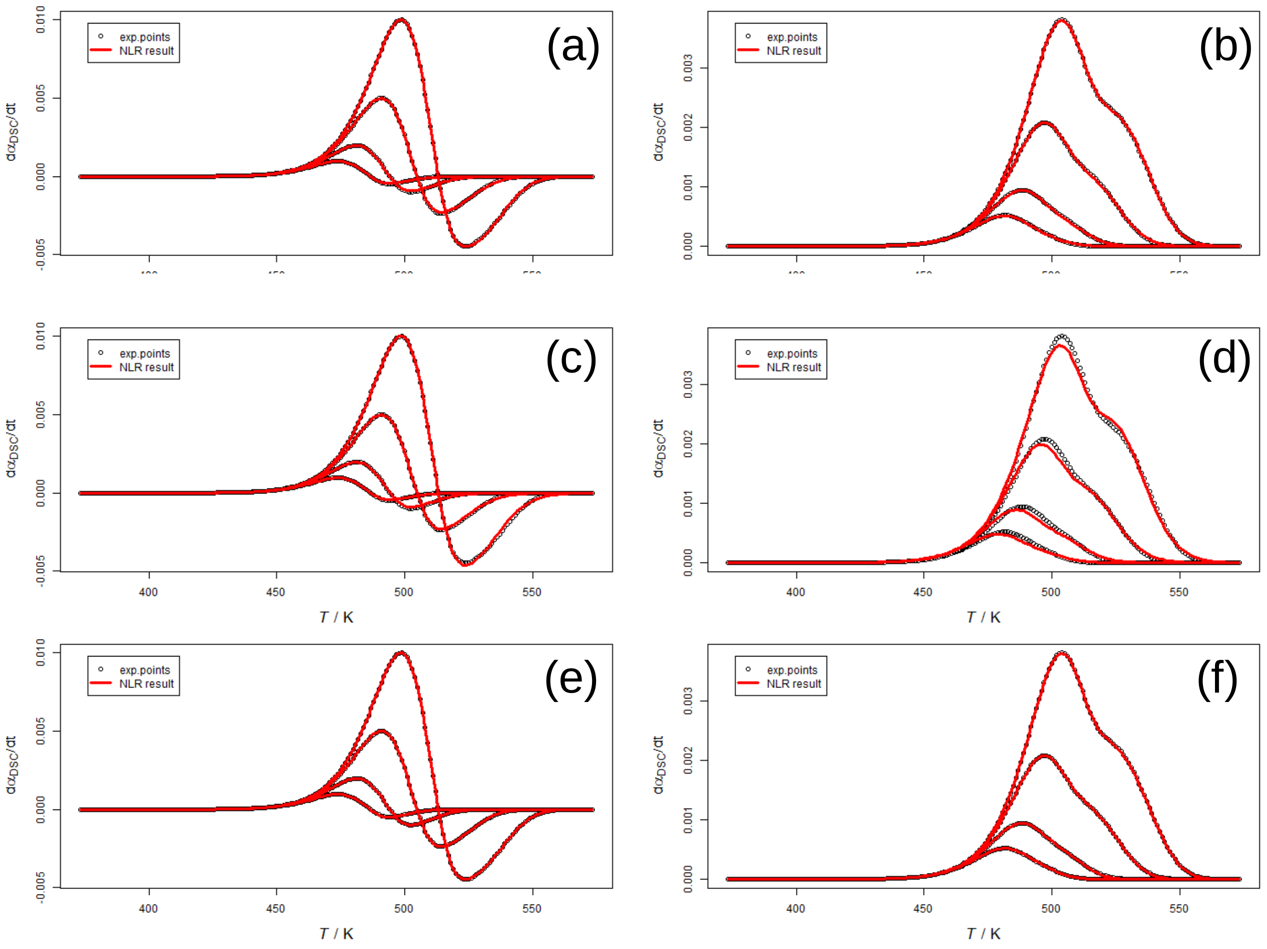

Figure S12. Formal kinetic and KDA fit (red lines) of data for Case 3 (points). (a) fit of DSC data with two independent Bna reactions, (b) fit of DTG data with two independent Bna reactions, (c, d) fit of DSC and DTG data with two independent Bna reactions, (e, f) fit of DSC and DTG data with two consecutive Bna reactions.

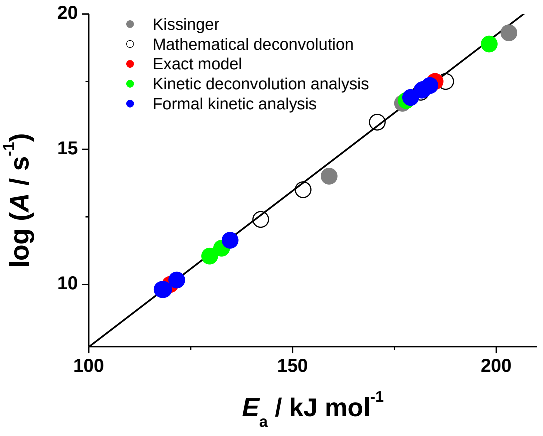

Figure S13. Linear dependence between lg$A$ and E_a observed for the kinetic parameters calculated using various methods for the simulated kinetic rate data (Case 3).
